# Supplementary material for: A Honeycomb-like Ammonium-Ion Fiber Battery with High and Stable Performance for Wearable Energy Storage
Source: Polymers (Basel). 2022 Oct 3;14(19):4149. doi: 10.3390/polym14194149 (PMC9573061; doi:10.3390/polym14194149)
Supplement: Supplementary file 1 [file polymers-14-04149-s001.zip › polymers-1908161-supplementary.pdf]

Supporting Information

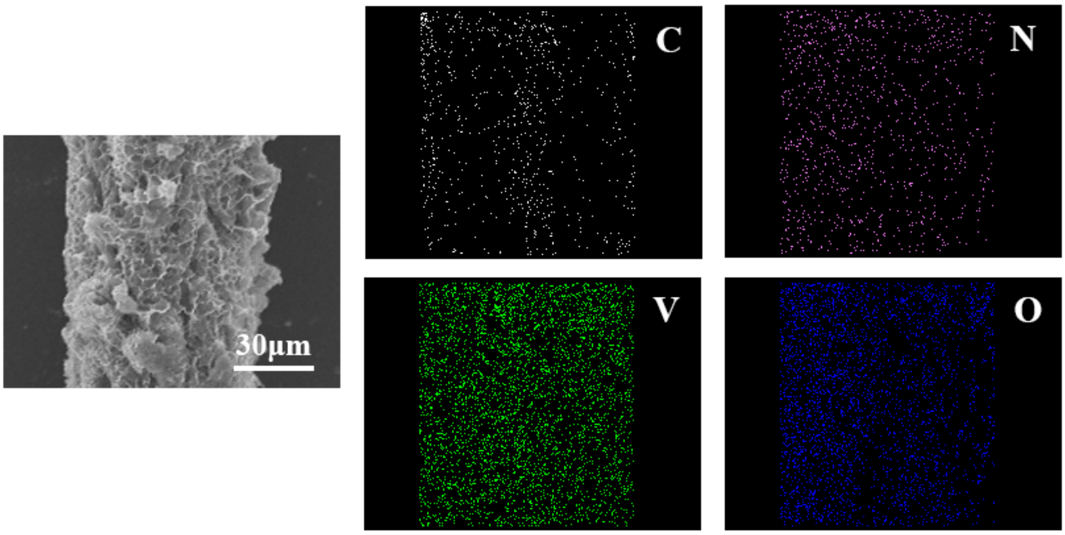

Figure S1: SEM images of $NH_4V_4O_{10}$@CNT fiber and EDS mapping of C, N, V, O, respectively

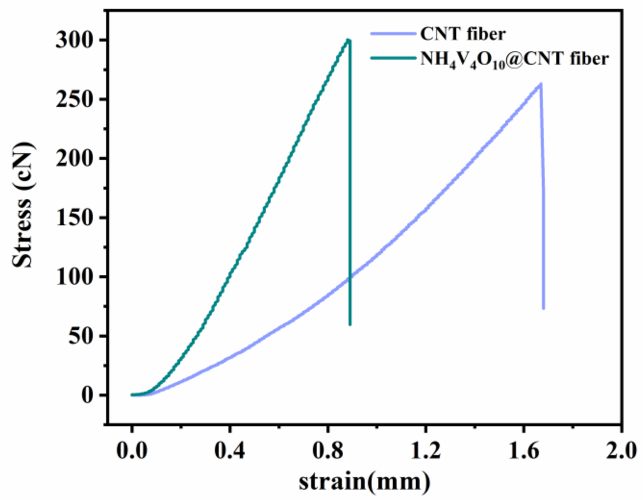

Figure S2: the stress-strain curve of CNT and $NH_4V_4O_{10}$@CNT

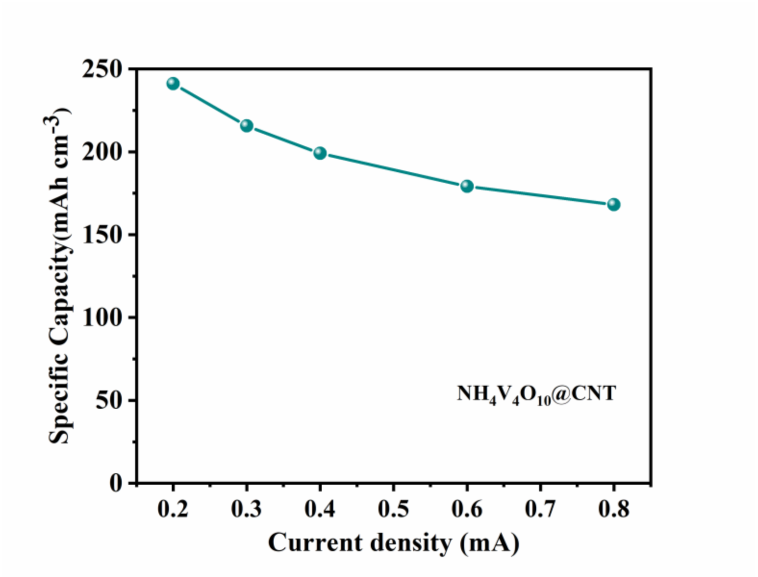

Figure S3: the specific capacity of $NH_4V_4O_{10}$@CNT electrode at different current density

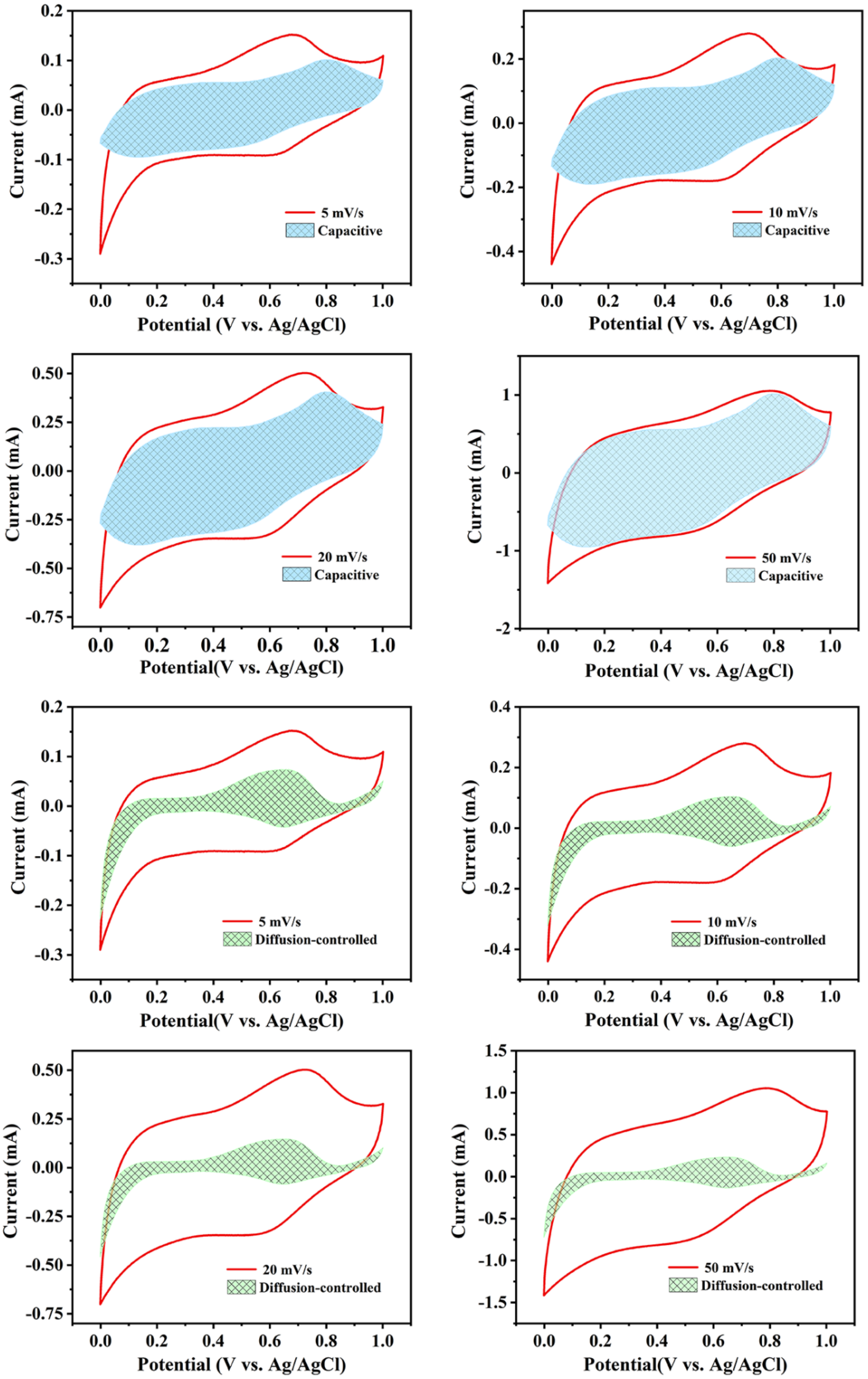

Figure S4: CV curves with the capacitive and pseudocapacitive fraction shown by the shaded area at different scan rate of NH4V4O10@CNT fiber battery

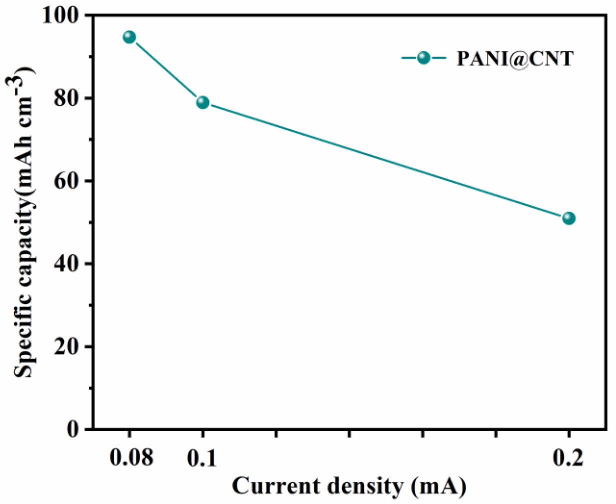

Figure S5: the specific capacity of PANI@CNT electrode at different current density

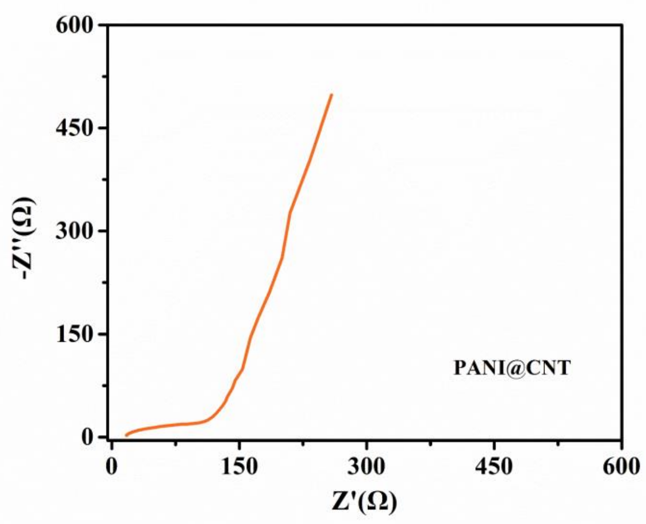

Figure S6: EIS of PANI@CNT fiber

$$(NH_4)_m(H_2O)NV_4O_{10} \leftrightarrows (NH_4)_{m-x}(H_2O)_{n-y}V_4O_{10}+xe^-+xNH_4^++yH_2O(0 \leqslant y \leqslant n)$$

Figure S7:The overall reactions in the charge and discharge processes of the full cell

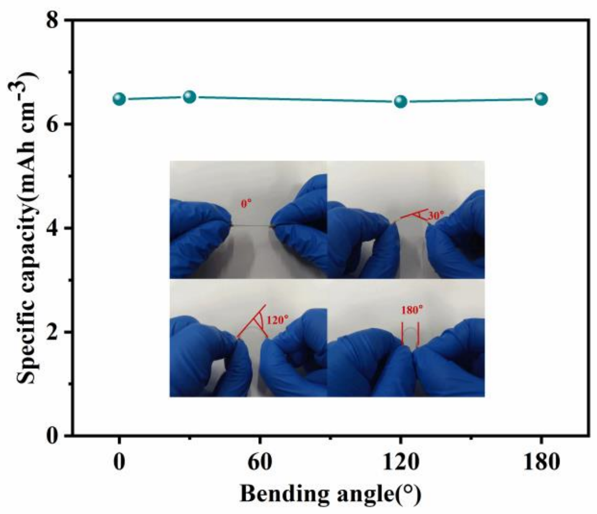

Figure S8: the specific capacity of the full cell under different bending states

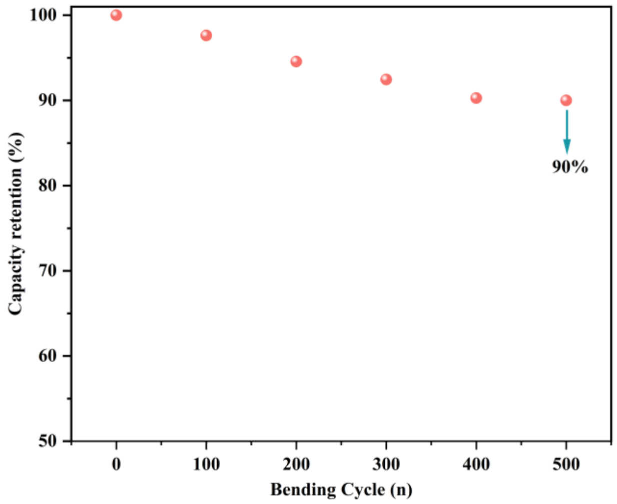

Figure S9: the capacity retention of the full cell under 500 bending cycles

Table S1: Compared to previous work on electrochemical performance of fiber batteries

Cathode /anode	Battery Types	Capacity	Capacity retention/cycles/ current density	Ref.
$NH_4V_4O_{10}$@CNT	Half cell	241.06 mAh cm^{-3}	N/A	Our
PANI	Full cell	6.86 mAh cm^{-3}	72.1%/1000/0.5 mA	work
Ni-NiO	Full cell	237.8 uAh cm^{-3}	96.6%/10000/3.7	[33]
Zn			A/g	
CC-ZnO@C@Zn	Full cell	3 mAh cm^{-3}	82%/1600/250 mA	[34]
CC-CCH@CMO			cm^{-3}	
Co_3O_4/N-rGO	Full cell	0.5 mAh cm^{-3}	NA	[35]
Zn				
Co/Co-N-C	Full cell	0.17 mAh cm^{-3}	NA	[36]
Zn				
